# Autism and depression are connected: A report of two complimentary network studies

**DOI:** 10.1177/1362361319872373

**Published:** 2019-11-10

**Authors:** Barbara FC van Heijst, Marie K Deserno, Didi Rhebergen, Hilde M Geurts

**Affiliations:** 1 University of Amsterdam, The Netherlands; 2 Dr. Leo Kannerhuis, The Netherlands; 3 Amsterdam Public Health Research Institute, The Netherlands; 4 GGZ inGeest Specialized Mental Health Care, The Netherlands

**Keywords:** autism spectrum disorders, depression, mastery, network analysis, psychiatric comorbidity, worry

## Abstract

Autism and depression often co-occur. Through network analysis, we seek to gain a better understanding of this co-occurrence by investigating whether (1) autism and depression share overlapping groups of symptoms and/or (2) are connected through a bridge of mastery or worry symptoms. This is addressed in two complimentary studies: (1) Study 1 focusing on depressed (*N* = 258) and non-depressed adults (*N* = 117), aged 60–90 years; (2) Study 2 focusing on autistic (*N* = 173) and non-autistic adults (*N* = 70), aged 31–89 years. Self-report questionnaire data were collected on autistic traits (AQ-28), depression symptoms (Study 1: Inventory of Depressive Symptomatology Self Report; Study 2: Symptom Checklist 90–Revised depression subscale), worry (Worry Scale-R) and mastery (the Pearlin Mastery Scale). For both studies, data were analysed by creating glasso networks and subsequent centrality analyses to identify the most influential variables in the respective networks. Both depressed and autistic adults are highly similar in the perceived amount of worries and lack of control. While caution is needed when interpreting the pattern of findings given the bootstrapping results, findings from both studies indicate that overlapping symptoms do not fully explain the co-occurrence of autism and depression and the perception of having control over your life, that is, mastery seems a relevant factor in connecting autism and depression.

It is well documented that people with an autism^1^ diagnosis have a high incidence of co-occurring mental health conditions, and depression is among the most reported (e.g. Ghaziuddin, Ghaziuddin, & Greden, 2002; Hofvander et al., 2009; Lever & Geurts, 2016). Before autistic adults receive their autism diagnosis, depression is one of the major first concerns expressed by autistic adults when contacting mental health care services (Geurts & Jansen, 2012; Jones et al., 2014). Recent large studies show that the prevalence of a lifetime depression diagnosis is up to 40.2% among autistic adults (Croen et al., 2015; Hudson, Hall, & Harkness, 2018). In addition, adults with a history of depression report a greater number of autistic traits than adults without a history of depression, 31% and 2.6%, respectively (Geurts, Stek, & Comijs, 2016). Furthermore, in the general population, depression and autistic traits are associated: people with more autistic traits show significantly more depression symptoms than those with less autistic traits (Kanne, Christ, & Reiersen, 2009), and autistic traits and depression are reported to be correlated (*r* = 0.27; Liew, Thevaraja, Hong, & Magiati, 2015). Hence, autism and depression seem to be interrelated, but it is still unknown why this co-occurrence happens. Depression might have same etiological origin as autism, share overlapping symptoms and/or can be the result from living with autism. In this article, we will report on two studies in which we focus on the relationship between autistic traits and depression symptoms in both a depression population (Study 1) as well as an autism population (Study 2).

The co-occurrence of two conditions (i.e. comorbidity) can be examined from different theoretical approaches: the latent variable approach and the network approach (Cramer, Waldorp, van der Maas, & Borsboom, 2010). In latent variable theory, the central idea is that depression and autism are latent variables that are measured through a number of symptoms, such as depressed mood and challenges in social communication. Comorbidity is then hypothesized to arise from a correlation between the latent variables. Explanations for comorbidity include, for example, a genetic disposition or a general predisposition towards negative affect that causes both latent variables (Borsboom, Cramer, Schmittmann, Epskamp, & Waldorp, 2011). Thus, when using this model, a common assumption is that the co-occurrence of two conditions such as autism and depression is caused by an overlap in etiological origin of both autism and depression. In the network approach, this assumption is not needed. The central idea is that mental health conditions are networks of interrelated symptoms that may cluster together in a meaningful way, but are not necessarily caused by a similar underlying latent variable. In this case, comorbidity is hypothesized to arise from direct symptom-to-symptom relations within and across diagnostic categories. These cross-disorder interrelations could be explained by overlapping symptoms (American Psychiatric Association (APA), 2013; Cramer et al., 2010). Within the network framework, an overlapping symptom is considered a *bridge symptom.* In the case of depression in autistic adults, it is argued that – on one hand – some autistic behaviours can be understood as signs of depression such as social withdrawal and flat affect while someone is not depressed (e.g. Ghaziuddin et al., 2002; Stewart, Barnard, Pearson, Hasan, & O’Brien, 2006). On the other hand, such behaviour can also be ignored as signs of depression as it is just attributed to being autistic (e.g. Ghaziuddin et al., 2002; Stewart et al., 2006). Social withdrawal and flat affect can in the network model be considered bridge symptoms as they link autistic behaviours to depression symptoms. Using the network approach, one can test whether autism and depression indeed share (groups of) overlapping symptom which in turn might explain the observed co-occurrence.

The network approach also provided the opportunity to test whether other factors, which are not symptoms or traits of the conditions themselves, act as bridge symptoms between two conditions. Based on the depression literature, we will test whether factors that reveal how people cope with stress will gain insight into the co-occurrence of autism and depression. It is widely reported that external stressors are risk factors for depression, such as stressful life events (Brilman & Ormel, 2001; Comijs et al., 2007) or interpersonal, financial and health-related stressors (Moos, Schutte, Brennan, & Moos, 2005). However, not just the events themselves but also how one can cope with stress is of importance in the relationship between stress and depression (e.g. Marshall & Lang, 1990; Steunenberg, Beekman, Deeg, Bremmer, & Kerkhof, 2007). Two intrapersonal factors of coping with stress that are hypothesized to be relevant are worry and mastery. Worry is defined as a so-called dysfunctional cognitive emotion regulation strategy of dealing with stressors (Wisocki, 1988). Worry has been found to correlate with depressive symptoms, *r* = 0.56 (Liew et al., 2015). Mastery is defined as the extent to which individuals consider that they are in control of their own lives, in contrast to being victims of fate (Pearlin & Schooler, 1978). A strong sense of mastery implies an internal locus of control is negatively correlated with depression (Marshall & Lang, 1990) and is predictive of recovery from late-life depression (Steunenberg et al., 2007). A low sense of mastery has been found to correlate with a decrease in emotional well-being, and specifically with depression (Bengtsson-Tops, 2004; De Beurs et al., 2005). Both intrapersonal factors have hardly been studied in autistic adults. In one study, it was reported that worry is (moderately) correlated with autistic traits (*r* = 0.34; Liew et al., 2015). In another study, it was shown that autistic adults’ perceived control over stimuli impacts their reported sensory sensitivity (Robertson & Simmons, 2015). Especially, how stressful one perceives life, that is, how difficult to master and cope with life’s stressors, seems to have an impact on quality of life of autistic adults (Bishop-Fitzpatrick, Minshew, Mazefsky, & Eack, 2017). Yet, to our knowledge, mastery has not been studied in autistic adults, while, as aforementioned, in the general population, this is considered to be an important intrapersonal factor associated with depression. We hypothesize that worry and mastery are of importance when linking autistic traits to depression as (1) how people cope with stress is an important predictor for the depression development and (2) autistic adults report high levels of stress (Gillot & Standen, 2007) and experience many stressful life events (e.g. Bishop-Fitzpatrick et al., 2017) which, in children, have been linked to the occurrence of depression (Ghaziuddin et al., 2002). Therefore, in two separate studies, we will test this hypothesis that worry and mastery are intrapersonal factors which link (i.e. bridge) autistic traits to depression symptoms using network analyses.

In sum, in this article, we will examine the co-occurrence of autism and depression with the network approach in two complimentary studies. The network approach provides the possibility to investigate the connectivity of specific characteristics of the two conditions and to pinpoint bridges between the conditions. With this method, we can disentangle whether (1) autism and depression share overlapping groups of symptoms (i.e. relationship among subscales of questionnaires) and/or (2) are connected through a bridge of mastery or worry symptoms. In the first study, we will focus on people diagnosed primarily with a depression (i.e. depression cohort), and in the second study, we will focus on people primarily diagnosis with autism (i.e. autism cohort).

## Method – Study 1: depression cohort

### Participants: depression cohort

In the Netherlands study of depression in older persons (NESDO), 510 depressed and non-depressed older individuals (60–93 years old) were recruited in a multi-site naturalistic prospective cohort study, to examine late-life depression in older adults. The NESDO sample and study design have previously been described (for a detailed description, see Comijs et al., 2011). Participants were recruited from mental health care institutes and general practitioners. Participants were excluded if they had a diagnosis of dementia, were suspected for dementia, had a Mini Mental State Examination (MMSE) score under 18 (please note that none of the participants had a MMSE score under 21), or had insufficient understanding of the Dutch language. The Autism Spectrum Questionnaire (AQ-28), which measures autistic traits, was added to the NESDO study after baseline measurement, between 2011 and 2012. All participants who had completed the AQ-28 and met the inclusion and exclusion criteria for the depression group were included in this study (*N* = 258; please note that this is the largely the same group of participants as studied in Geurts et al., 2016).

#### Depression group

These participants (*N* = 258, aged 60–90 years, see Table 1) had a primary diagnosis of major depression, dysthymia or depressive disorder not otherwise specified (DD-NOS, a.k.a. minor depression) according to the *Diagnostic and Statistical Manual of Mental Disorders* (4th ed.; DSM-IV; APA, 2000), or a positive screening with the Geriatric Depression Scale (GDS-15) combined with meeting the criteria for a current depression at baseline measurement. The Composite International Diagnostic Interview (CIDI; Wittchen et al., 1991) was used to assess the presence of mood disorders. In the past year before baseline measurement, 26% of participants met criteria for dysthymia (*N* = 68) and 94% for major depression (*N* = 243). In the past month before baseline, 6% of participant met criteria DD-NOS (*N* = 16). Some participants met criteria for both dysthymia and major depression.

**Table 1.** Participant characteristics for the depression cohort (Study 1) and the autism cohort (Study 2).

Gender (M/F)	*N* = 258	Depression cohort – Study 1			Statistics	*N* = 173	Autism cohort – Study 2			Statistics
		Depression	*N* = 117	Comparison			Autism	*N* = 70	Comparison	
		83/175		44/73	χ^2^(1) = 1.06, *p* = 0.30		101/71^a^		40/30	χ^2^(2) = 0.457, *p* = 0.796
		M (SD; range)		M (SD; range)			M (SD; range)		M (SD; range)	
Age	258	70.1 (7.2;60–90)	117	69.4 (6.5;60–85)	*F*(1, 373) = 0.80, *p* = 0.37, η^2^ = 0.002	173	53.7 (12.2;31–89)	70	56.2 (10.3;34–79)	*F*(1, 243) = 2.24, *p* = 0.14, η^2^ = 0.009
Social Skills	250	15.3 (4.5;7–28)	114	11.8 (3.3;7–20)	*F*(1, 362) = 55.76, *p* < 0.001, η^2^ = 0.13	171	21.5 (3.7;13–28)	69	13.4 (3.7;7–23)	*F*(1, 240) = 241.3, *p* < 0.001, η^2^ = 0.50
Routine	255	9.8 (2.6;4–16)	117	7.5 (1.8;4–12)	*F*(1, 370) = 74.10, *p* < 0.001, η^2^ = 0.17	171	12.1 (2.3;5–16)	70	7.4 (2.2;4–14)	*F*(1, 241) = 216.4, *p* < 0.001, η^2^ = 0.48
Switching	257	10.5 (2.8;4–16)	115	7.8 (2.4;4–13)	*F*(1, 370) = 76.15, *p* < 0.001, η^2^ = 0.17	172	13.0 (2.4;5–16)	70	8.0 (2.2;4–13)	*F*(1, 242) = 221.8, *p* < 0.001, η^2^ = 0.48
Imagination	245	19.3 (3.6;10–31)	115	17.2 (3.5;9–25)	*F*(1, 358) = 28.46, *p* < 0.001, η^2^ = 0.07	166	22.8 (4.7;10–32)	70	15.1 (3.4;8–23)	*F*(1, 236) = 151.3, *p* < 0.001, η^2^ = 0.39
Numbers	252	8.9 (3.4;5–20)	113	9.3 (3.4;5–18)	*F*(1, 363) = 0.80, *p* = 0.37, η^2^ = 0.002	170	13.3 (4.1;5–20)	70	8.5 (3.5;5–18)	*F*(1, 240) = 76.0, *p* < 0.001, η^2^ = 0.24
Mood	254	8.7 (5.0;0–21)	114	1.2 (2.0;0–14)	*F*(1, 366) = 245.80, *p* < 0.001, η^2^ = 0.40	−		−		−
Motivation	250	4.9 (3.1;0–13)	112	0.6 (1.2;0–9)	*F*(1, 360) = 200.55, *p* < 0.001, η^2^ = 0.36	−		−		−
Somatic	255	9.6 (4.3;0–22)	114	4.6 (3.0;0–15)	*F*(1, 367) = 124.98, *p* < 0.001, η^2^ = 0.25	−		−		−
Depression	−		−		−	157	32.4 (12.1;16–76)	67	19.5 (4.7;16–44)	*F*(1, 224) = 71.8, *p* < 0.001, η^2^ = 0.24
Social	242	3.8 (4.1;0–19)	113	0.6 (1.9;0–17)	*F*(1, 353) = 59.36, *p* < 0.001, η^2^ = 0.14	172	4.9 (4.7;0–19)	69	1.0 (1.7;0–11)	*F*(1, 241) = 45.7, *p* < 0.001, η^2^ = 0.16
Financial	231	2.5 (4.1;0–20)	110	0.8 (2.1;0–12)	*F*(1, 339) = 15.77, *p* < 0.001, η^2^ = 0.04	168	4.1 (4.4;0–19)	68	2.0 (3.4;0–20)	*F*(1, 236) = 11.6, *p* = 0.001, η^2^ = 0.05
Independent	241	7.0 (4.7;0–20)	112	3.7 (4.0;0–17)	*F*(1, 351) = 42.94, *p* < 0.001, η^2^ = 0.11	173	5.8 (4.5;0–19)	68	3.7 (3.1;0–16)	*F*(1, 241) = 12.6, *p* < 0.001, η^2^ = 0.05
Mastery	238	16.7 (4.4;5–25)	113	8.6 (3.2;5–20)	*F*(1, 349) = 233.48, *p* < 0.001, η^2^ = 0.40	172	15.9 (4.0;5–25)	69	9.6 (3.0;5–18)	*F*(1, 241) = 139.1, *p* < 0.001, η^2^ = 0.37

SD: standard deviation.

a Participants were asked to report their biological sex and could chose between male, female, or other. One person marked other.

#### Comparison group

Participants (*N* = 117, aged 60–85 years) were recruited via general practitioners in the Netherlands. Inclusion criteria were as follows: a negative screening with the GDS-15 and no lifetime diagnosis of depression or dementia. None of these participants met criteria for a mood disorder as measured with the CIDI at baseline.

The study protocol of NESDO has been approved by the Ethical Review Board of the VU University Medical Centre, and subsequently by all participating clinical institutes. Written informed consent was obtained from all participants.

### Materials

#### Autistic traits

The abridged version of the Dutch Autism Spectrum Questionnaire (AQ-Short; Hoekstra et al., 2011) is a 28-item self-report questionnaire. The items assess five domains of cognitive strengths and difficulties related to autism, corresponding to the subscales: (1) poor social skills (‘Social Skills’), (2) a desire for routine (‘Routine’), (3) difficulty in switching between tasks (‘Switching’), (4) impaired imagination (‘Imagination’) and (5) a fascination for numbers and patterns (‘Numbers’). Items include, for example, ‘I find it difficult to work out people’s intentions’. Some items are recoded so that a higher score indicates more autistic traits. The internal consistency (α = 0.77/0.86) and validity of the AQ-28 are acceptable to good (Hoekstra et al., 2011).

#### Depression symptoms

The IDS-SR (Rush, Gullion, Basco, Jarrett, & Trivedi, 1996) is a 30-item self-report questionnaire with statements related to key symptoms of depression. Previously, in a sample of older adults, factor analysis identified three factors corresponding to the subscales: (1) mood, related to feelings of sadness, (2) motivation, related to apathy and (3) somatic or vegetative symptoms (Hegeman et al., 2012). The statements refer, for example, to feelings of sadness or loss of energy. A higher score indicates more severe depressive symptomatology. The internal consistency of the three subscales ranges from α = 0.70 to α = 0.93 (Hegeman et al., 2012). The psychometric properties of this self-report scale are sound (Corruble, Legrand, Duret, Charles, & Guelfi, 1999).

#### Worry

The Worry Scale-R is a shortened 15-item self-report questionnaire from the 88-item Worry Scale for Older Adults (Wisocki, 1988). The items assess three domains of worries, corresponding to the subscales: (1) worries about loss of independence (‘Indep’), (2) worries about social conditions (‘Social’) and (3) financial worries (‘Financial’). Statements include, for example, ‘It worries me that I will have to be taken care of by my family’. A higher score indicates more worries. Internal consistency is good, α = 0.85 (Van der Veen, Comijs, van Zelst, Schoevers, & Oude Voshaar, 2014).

#### Mastery

The Pearlin Mastery Scale (Pearlin & Schooler, 1978) is a five-item self-report questionnaire that measures perceived control over life’s stressors. Items include, for example, ‘There is little I can do to change many of the important things in my life’. A higher score indicates a lower sense of mastery. The original version of Pearlin and Schooler consists of seven items, including two positive-worded items. In our study, we used the shortened five-item version, with only negative items, as this version was found to have better reliability, α = 0.71/0.77 (Gadalla, 2009; Van der Zanden, Kramer, Gerrits & Cuijpers, 2012). The total score is called ‘Mastery’.

### Data analysis

In order to describe the studied population, we compared the depression group with a comparison group (see Table 1) on gender, age and the outcome measures. Continuous variables were tested with analysis of variance (ANOVA) and categorical variables with Pearson’s chi-square test.

First, for the main analysis, the network structure of the data was estimated in the depression group. We checked whether the variables of interest were normally distributed in order to determine which network package was the most appropriate. According to the Shapiro–Wilk test, none of the variables were normally distributed (see Supplementary Material Section 1 for distributions of each of the measures), thus we implemented a non-paranormal family of models (i.e. SCEPTIC) to accommodate the non-Gaussian distribution of the variables (i.e. Gaussian Graphical Model; GGM) to estimate a network based on regularized partial correlation coefficients. In order to be able to estimate the network using SCEPTIC, missing values needed to be imputed. We applied multivariate imputation by chained equations in the R-package *mice* based on predictive mean matching. These analyses were run using the *qgraph* package (Epskamp, Cramer, Waldorp, Schmittmann, & Borsboom, 2012) in the statistical programming language R, version 3.6.0 (R Core Team, 2018). This gave us a graphical depiction of the data, that is, a network with nodes and edges. The nodes represented the variables of interest, which were the aforementioned subscales of the AQ, IDS-SR, and worry questionnaires and the total score of the mastery questionnaire. Subscale scores were preferred over the use of item scores because this reduced the number of nodes, and, therefore, the number of estimations and error, while still being able to estimate a detailed pattern of interrelations (Deserno, Borsboom, Begeer, & Geurts, 2017). The edges represented partial correlations between these variables, after conditioning on all other variables in the data set. To control for false-positives and create a sparse and interpretable network, the graphical LASSO (least absolute shrinkage and selection operator) regularization with extended Bayesian information criterion (EBIC) model selection was used (for details, see the tutorial by Epskamp & Fried, 2018).

Second, centrality analyses were performed to identify the most important and influential variables in the network. Indices of strength (the sum of the absolute values of the number of edges connecting to a node) and betweenness (the number of times a node acts as a bridge along the shortest path between two other nodes) were calculated. In order to test the stability and robustness of the estimated network, we performed additional network stability checks using the R-package *bootnet* (Epskamp, Borsboom, & Fried, 2018) and appended the bootstrapping plots corresponding to the correlation stability (CS) coefficients reported in the result section as Supplementary Material. This coefficient can be interpreted as a metric quantifying the maximum proportion of cases that can be dropped until the probability of retrieving a correlation higher than 0.7 between the respective centrality coefficients and the original centrality coefficients will fall below the 95% threshold.

## Results – Study 1: depression cohort

### Sample

Overall the depression group reported more autistic traits (except on the *Numbers* scale), more worries and less mastery then the age-matched comparison group. For detailed descriptives of the sample and the statistics of the comparisons, please see Table 1.

### Network

**Figure 1.**
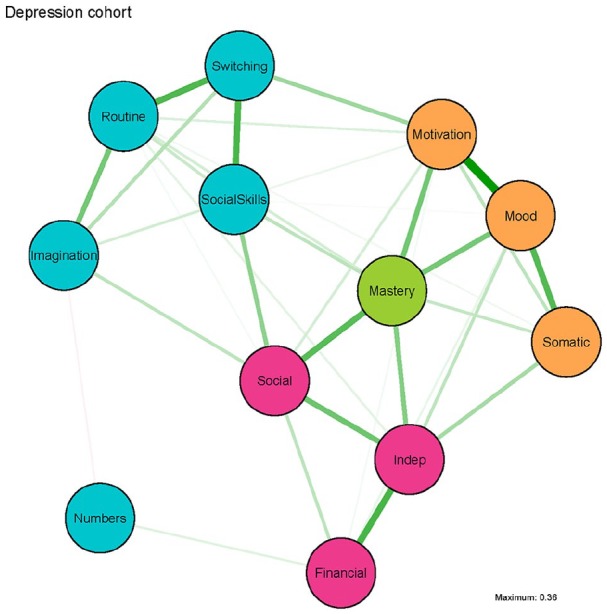
Network of autistic traits, depression symptoms, worry symptoms and mastery in Study 1: the depression cohort. Green lines represent positive associations and red lines represent negative associations. A thicker and more saturated edge represents a stronger connection, and nodes that are strongly interconnected are depicted closer together. Autistic traits measured by the AQ-28: Social Skills = poor social skills; Routine = a desire for routine; Switching = difficulty in switching between tasks; Imagination = impaired imagination; Numbers = a fascination for numbers and patterns. Depression symptoms measured by the IDS-SR: Mood; Motivation; Somatic. Worries measured by the Worry Scale-R: Social = worries about social conditions; Financial = financial worries; Indep = worries about loss of independence. Mastery measured by the Pearlin Mastery Scale: Mastery.

The network structure of the data was visualized in Figure 1. The graph showed that all associations were positive except for the relationship between *Numbers* and *Imagination*. These two autism trait domains do seem show a weak negative relationship. Moreover, autism traits clustered together, as well as depression and worry symptoms. Connections that were expected such as a close association between *Social Skills* and worries about social conditions (*Social*) were present. The *Numbers* node was only weakly connected to other nodes. The strongest direct connection between the autism trait nodes and depression symptom nodes was between *Switching* and *Motivation*. There are various other direct weak connections between autism nodes and depression nodes, but the stronger connections between these two were going via the *Mastery* node. *Mastery* seems to be a link between Depression and Worry nodes, although there are also direct (weaker) connections between these Depression and Worry nodes.

### Centrality

**Figure 2.**
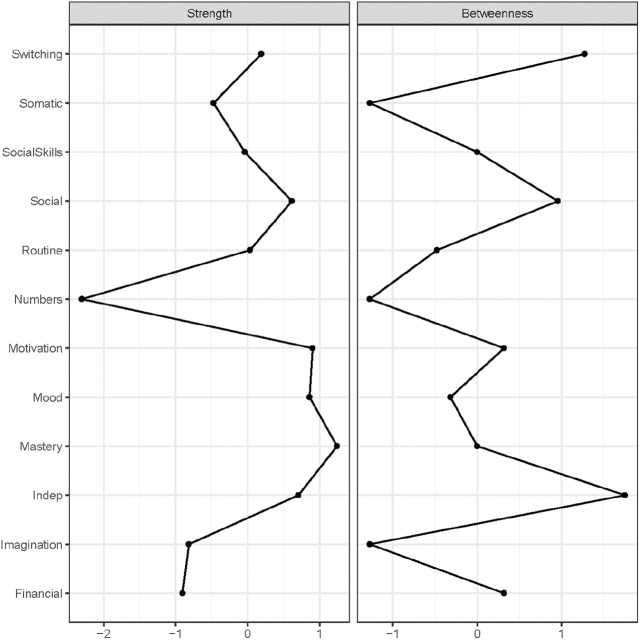
Standardized (i.e. *z*-scores) centrality indices node strength and betweenness for the depression cohort network in Figure 1.

*Mastery* had the greatest number of connections in the network, which was also visible in the graphical depiction of the network (Figure 1). *Numbers* was the least central node, which is not surprising considering it was only weakly connected to other nodes in the graphical depiction of the network. Worries about independence (*Indep*) ranks highest on betweenness (e.g. acts most often as a bridge on the shortest path between two other nodes). However, the stability checks revealed that for strength the CS coefficient (0.52) is just above the stability threshold of 0.5 (Epskamp et al., 2018), while betweenness is unstable (<0.05, see Supplementary Material). So, in Figure 2, we show the results for both strength and betweenness. However, given the stability findings and one should refrain from drawing strong conclusions related to betweenness but one can reliably interpret strength.

## Conclusion – Study 1: depression cohort

In line with previous research, there are more autistic traits, more worries and less perceived control in those that are known with a history of depression. Moreover, there is, as expected, a direct relation between autism and depression. Regarding the commonly hypothesized symptom overlap (Ghaziuddin et al., 2002; Stewart et al., 2006), we only observed that difficulties with switching between tasks is related to feeling less motivated. However, many connections between autism and depression seem funnelled through mastery (i.e. most connected node), but also other direct connections became apparent. Hence, the feeling of having control of your life is indeed of importance for the autism–depression relationship, but is not the single reason for this relationship.

As this network was solely constructed based on data of people with a depression diagnosis, but no known autism diagnosis, it does not yet fully help us in understanding how depression and autism are related in autistic individuals. From a dimensional perspective on psychopathology, a network of symptoms should be the same in different random samples. Groups of people will, from such a dimensional stand, not differ in the structure of the network of nodes, but solely on the strength in the connections between the nodes. However, first of all the sample of this study is not random, but selected on the presence of depression. Second, if a specific disorder and/or disability is considered a so-called taxon, another structure of a network can underlie the co-occurrence of two conditions. Groups of people will not solely differ in the strength of the connections between the nodes, but might also differ in the nodes that form bridge symptoms. Thus, a different network could occur in a different sample. Furthermore, the advisory board of autistic adults consulted for this research, suggested, after seeing the depression network, that the network may be different for autistic adults. Therefore, we ran a second complimentary study, analogous to Study 1, where we included participants with an autism diagnosis. Our hypothesis is that the observed network in Study 1, with a central role of mastery, will also be observed in an autism population.

## Method – Study 2: autism cohort

### Participants: autism group

Participants were recruited from two sources: (1) 167 adults with and without an autism diagnosis who previously participated in a study about aging and autism (for a detailed description see Lever & Geurts, 2016) and had given permission to be contacted for future research and (2) 78 adults with and without an autism diagnosis who were recruited with ads in an autism magazine and via social media. Two participants were excluded because they did not fill out the AQ completely. An IQ < 70 and a history of neurological disorders or schizophrenia were exclusion criteria. In total, 243 adults aged 31–89 years participated in this study. Please note that the participants in this study were younger than in Study 1.

#### Autism group

All these participants (*N* = 173) had a self-reported clinical diagnoses of autism which was formally assessed by a qualified health care professional. In addition, the autism diagnosis was verified for 47% of the participants (Lever & Geurts, 2016) with the Autism Diagnostic Observation Schedule module 4 (ADOS; De Bildt, Sytema, Meffert & Bastiaansen, 2016; Hus & Lord, 2014). Of these 81 participants, 23 scored below the cut-off for autism spectrum disorder (ASD; <7), 29 scored above the ASD cut-off but below the autism cut-off (<10) and 29 scored above the autism cut-off (⩾10). Self-reported psychiatric disorder was 19.6% for a current depression (*N* = 34) and 31.2% reported a previous depression (*N* = 54).

#### Comparison group

All participants (*N* = 70) neither had themselves nor an immediate family member with an autism, attention-deficit hyperactivity disorder (ADHD) or schizophrenia diagnosis. Self-reported psychiatric disorder was 0% for a current depression (*N* = 0) and 5.7% for a previous depression (*N* = 4).

The study was approved by the ethical review board of the Department of Psychology at the University of Amsterdam (2015-BC-4270 and 2018-BC-9285). Written informed consent was obtained from all participants.

### Materials

#### Autistic traits

The full version of the Autism Spectrum Questionnaire (Baron-Cohen, Wheelwright, Skinner, Martin, & Clubley, 2001), a 50-item self-report questionnaire, was administered. The subscales of the full version of the AQ and the abridged AQ version, which was used in Study 1, are not similar. Hence, for the sake of consistency, we only used the exact same 28 items as in Study 1 and calculated the same subscales as for the abridged version. For further details on the AQ-28, see the ‘Method – Study 1: depression cohort’ section.

#### Depression symptoms

The Symptom Checklist 90–Revised (SCL-90-R; Arrindell & Ettema, 2005; Derogatis & Cleary, 1977) is a 90-item self-report questionnaire that measures psychological distress. Participants rate how much the specific problem or complaint has distressed them in the past week including today. There are eight subscales focusing on a wide range of difficulties, but for this study, the subscale depression was used to serve as a proxy for depression. This depression subscale consisted of 16 items, for example, ‘Feeling blue’ or ‘Feelings of worthlessness’. A higher score indicated more problems. The depression scale had a high internal consistency, α = 0.91 (Arrindell, Barelds, Janssen, Buwalda & van der Ende, 2006).

#### Worry and mastery

Identical to Study 1, the Worry Scale-R (Wisocki, 1988) and Pearlin Mastery Scale (Pearlin & Schooler, 1978) were administered, for details see Study 1.

### Data analysis

The exact same analyses as in Study 1 were carried out to optimize consistency. Please note that this network, however, consisted of 10 nodes, 2 nodes less than in Study 1. This was due to the inclusion of another depression questionnaire, resulting in a different number of indicators for depression (see the ‘Material’ section). Analogous to Study 1, we again tested whether the measures were normally distributed (see Supplementary Material). As none of the measures were normally distributed, we followed the same procedure as in Study 1.

Finally, statcheck (Epskamp & Nuijten, 2016) was run on all results in this article (i.e. of Study 1 and Study 2) to check for errors in statistical reporting.

## Results – Study 2: autism cohort

### Sample

Overall, the autism group reported more depressive symptoms, more worries and less mastery than the age-matched comparison group. For detailed descriptives of the sample and the statistics of the comparisons, please see Table 1.

### Network

**Figure 3.**
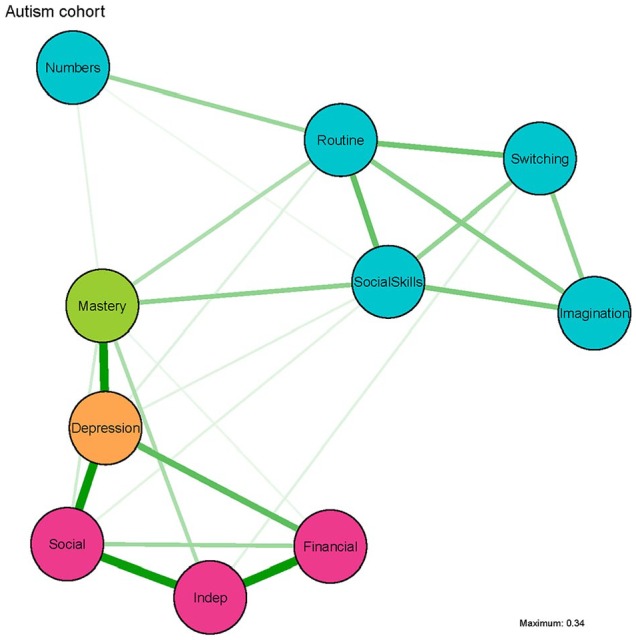
Network of autistic traits, depression symptoms, worry symptoms and mastery in Study 2: the depression cohort. Green lines represent positive associations and red lines would represent negative associations, but are not present in this network. A thicker and more saturated edge represents a stronger connection, and nodes that are strongly interconnected are depicted closer together. Autistic traits measured by the AQ-28: Social Skills = poor social skills; Routine = a desire for routine; Switching = difficulty in switching between tasks; Imagination = impaired imagination; Numbers = a fascination for numbers and patterns. Depression symptoms measured by the SCL-90 sub scale for depression: Depression; Worries measured by the Worry Scale-R: Social = worries about social conditions; Financial = financial worries; Indep = worries about loss of independence. Mastery measured by the Pearlin Mastery Scale: Mastery.

As in the depression cohort network (Figure 1), we saw only positive associations (Figure 3). Again the autistic traits clustered together, although *Numbers* was, like in Study 1, slightly separated from the other autistic traits nodes. There was only one node for both depression and mastery, so no clustering was possible in these domains. Worries about finances (*Financial*), and especially worries about social conditions (*Social*), and *Depression* were strongly interconnected. Also, in contrast with Study 1, there was no strong path from the autism node *Social Skills* to the Worry node *Social*. The direct links between *Depression* and the autistic traits nodes were relatively weak. The autism nodes *Routine* and *Social Skills* have a relatively strong connection with *Mastery*, which in turn has a strong connection to the *Depression* node.

### Centrality

**Figure 4.**
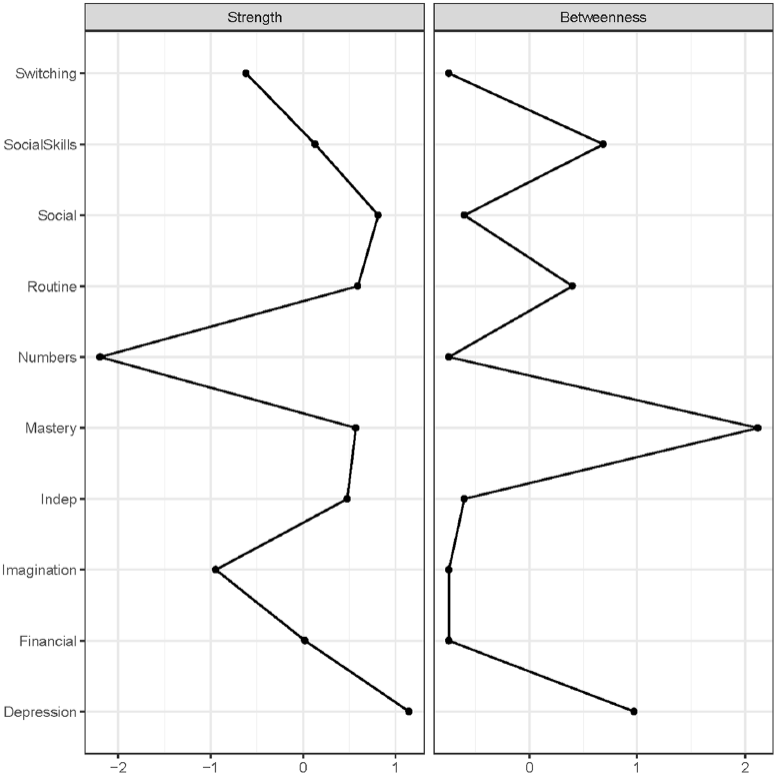
Standardized (i.e. *z*-scores) centrality indices node strength and betweenness for the autism cohort network in Figure 3.

The strength centrality measure (Figure 4) suggests that *Depression* has the most connections in the network, directly followed by worries about social situations (*Social*). *Numbers* was the least central node. Mastery ranks the highest on betweenness (e.g. acts most often as a bridge on the shortest path between two other nodes). However, stability checks revealed that for strength, the CS coefficient (0.44) is just below the stability threshold of 0.5, while betweenness is again unstable (0.13, see Supplementary Material), thus the same cautiousness as in Study 1 is warranted.

## Conclusion – Study 2: autism cohort

Similar to Study 1, autism traits cluster together. If we focus solely on the symptom relationships, it seems that these relations are relatively weak when we take into account the relationship among all other factors in the presented network. However, as depression is here just one single scale, we might lack the needed psychometric resolution to get a more in-depth picture of the potential symptom overlap between depression and autism. When focusing on the intrapersonal factors, mastery seems to play a central role in the network connecting depression with autism traits as hypothesized and observed in Study 1. However, as the network metrics are considered rather unstable, we can unfortunately not draw a firm conclusion on whether mastery indeed acts as a bridge between the autism traits and depression. The underlying assumption of the hypothesis that both Worry and Mastery were relevant interpersonal factors was that autistic adults might experience more worries and less control, as has been reported for people experiencing depression (Bengtsson-Tops, 2004; De Beurs et al., 2005). We do indeed find that autistic adults report more worries and do feel less in control of their own lives and more as victims of fate (i.e. low mastery) as compared to the age-matched control group. However, whether this is as severe as what we observe in the depressed individuals is unclear. In order to explore whether autistic participants differ from depressed individuals, we ran additional exploratory analyses combining data of Study 1 and Study 2.

### Exploratory analyses across Study 1 and Study 2

#### Statistical analyses

In order to explore the differences in worry and mastery between the two clinical groups across both studies and a comparison group, we combined the comparison groups into one comparison group. In this way, we are able to determine whether there is an actual difference between the clinical groups or whether observed potential differences are due to the different age-ranges of both clinical groups (Study 1: 60–93 years; Study 2: 31–89 years). A one-way multivariate analysis of covariance (MANCOVA) was performed with group as the independent variable (three levels), the worry and mastery scales as dependent variables and age as a covariate. Please note that the family of *t*-test and *F*-test violations of normality we reported in both Study 1 and Study 2 are not a problem for the validity of the model when a sample size exceeds 50 (see Pituch & Stevens, 2015).

#### Results group comparisons

After listwise deletion for missing variables, we could include 225 participants in the depression group, 166 in the autism group and 176 in the comparison group. There was a significant effect of group on the combined outcome variables after controlling for age, *F*(8, 1120) = 41.94, *p* < 0.001, Wilks’ Λ = 0.592. The autism and depression group did not statistically differ (*p* > 0.05) from each other on all four outcome measures, and both groups had higher scores on the worry and mastery scales than the combined comparison group. Age only had a significant effect on social (*p* = 0.044) and financial worries (*p* < 0.001), with lower age corresponding to more worries. More detailed results (including descriptives) can be found in Supplementary Material.

#### Conclusion group comparisons

This pattern of findings suggest that both clinical groups seem to experience similar amount of worries and lack of mastery, which is much higher than typically observed in people without any of these diagnoses. So, also in a group of autistic adults, feelings of a low sense of control and worries about social relationships, finances and independency are clearly present and similar to individuals who are depressed. Moreover, this pattern of findings cannot be fully explained by the differences in age-ranges between the groups.

## Discussion

In order to improve the understanding of the commonly reported co-occurrence of autism and depression (Hudson et al., 2018), we used network analyses to determine how autism- and depression-related behaviours are intertwined and if the way people cope with stress can provide a new perspective on this relationship. Our findings show that this co-occurrence cannot be simply attributed to overlapping groups of symptoms of autism or depression itself. Simultaneously, we can also not unequivocally conclude that the perceived control over life, that is, mastery, serves as the most important bridge between the symptom clusters of autism and depression. In line with the previously postulated hypothesis, mastery is, however, strongly related to many autism and depression behaviours for both clinical groups. In fact, mastery seems to bridge some of the connections between autism and depression, but is not the single relevant bridge as there are also some direct connections between autism and depression.

Besides mastery, worry was considered as a bridge between autism and depression. Worry is considered to be closely linked to depressive symptoms – it is even part of the symptoms in the *Diagnostic and Statistical Manual of Mental Disorders* (5th ed.; DSM-5) to specify depression with anxious distress (i.e. difficulty concentration because of worry, fear that something awful may happen). This is also what we observe in this study. Interestingly, while in the depression group, social worries were related to self-reported social skills, this relationship was less evident in the autism group. Autistic adults did report social difficulties and social worries, but the strongest link between these two went through feeling in control and feeling depressed. This might imply that social worries are specifically relevant for depressed feelings and not so much the actual self-perceived social skills. Nonetheless, in order to determine whether there are indeed causal relationships, longitudinal or intervention studies are needed.

This study is the first study using network theory to try to unravel the relationship between autism and depression by focusing on intrapersonal factors which are relevant for dealing with stress. Both intrapersonal factors – Worry and Mastery – gain research attention in the depression literature, but are, so far, largely ignored in autism research. We show that autistic adults do not just experience worries about their social conditions, finances and about loss of independence but also feel that they are not in control. In this respect, they are even indistinguishable from the group of depressed individuals.^2^ Especially, social worries and financial worries seem to increase with age, as age does have an impact on the amount of worries in both the depressed and autistic group of individuals.

As we conducted two complimentary but independent studies, this limits the direct comparability of the two networks. This lack of comparability is due to the difference in depression questionnaires used, the difference in the number of nodes and differing age-ranges of the people included. The data used in this article were part of two larger ongoing longitudinal studies that had overlapping questionnaires, but were, unfortunately, not completely identical. Depression was measured with the IDS-SR (Rush et al., 1996) depression cohort and with a subscale of SCL-90-R (Arrindell & Ettema, 2005) in the autism cohort. When measuring the construct of depression, even different questionnaires should in principle measure the same construct. The SCL-90-R depression subscale has been found to have good convergent validity with the depression diagnosis, and with other depression scales such as the Beck Depression Inventory (Brophy, Norvell, & Kiluk, 1988; Koeter, 1992). Given the aforementioned findings, it is likely that this also counts for the relation between the IDS-SR and the SCL-90-R subscale. Therefore, the difference in used questionnaires does not necessarily hinder comparability, but the differing number of nodes in the networks invalidates a direct comparison (IDS-SR subscales correspond with three nodes; SCL-90-R depression subscale with only one node).

An additional (associated) challenge, however, is related to the discussion whether standard depression measures are actually suitable for autistic adults as some might experience difficulties with articulating one’s own internal experience (Cassidy, Bradley, Bowen, Wigham, & Rodgers, 2018). Whether the mode of assessment and the language used in the SCL-90 depression scale is appropriate for autistic adults has not been tested yet. However, as this is the case for the majority of depression measures (Cassidy et al., 2018), it remains unclear whether another measure might have been more valid to assess depressive symptoms in autistic adults.

Another limiting factor in comparing the two networks is potentially that the autism group is younger than the depression group. It is, for example, known that older people cope with stressors in a different way than younger adults as older age has been found to be a predictor of a lower sense of mastery (Smits & Bosscher, 1998). However, our findings suggest that this effect is not strongly present in the studied cohorts. As aforementioned, age is a relevant factor, though, for social and financial worries.

In sum, the two complimentary studies to investigate the often reported co-occurrence of autism and depression by studying the respective networks expand the field towards a better understanding of the co-occurrence of autism and depression. The reported networks suggest that how one deals with stress is a relevant factor to take into account. Nonetheless, while causal pathways are not yet identified, it is clear that both depressed adults as well as autistic adults report more worries and a lower sense of mastery. The latter is connected to both autism and depression symptoms, although we cannot conclude that it is the solely relevant bridge symptom. The fact that feelings of mastery are low is of relevance for clinical practice. Mastery is generally seen as a personality trait, and there are indicators that mastery can improve over time with a decrease in depressive symptoms (Bengtsson-Tops, 2004; Deeg & Huisman, 2010) and with intervention (Van der Klink, Blonk, Schene & van Dijk, 2001, 2003; Van der Zanden et al., 2012). Mastery could be a valuable target in interventions, for both depressed individuals and for autistic individuals. The lack of perceived mastery can also be used as an indicator to signal vulnerability for developing additional problems. If one believes to be in control when coping with stress, this could potentially be one mechanism to reduce depressive symptoms. The autism population is especially vulnerable to stressful situations, because of mental and physical health issues (Croen et al., 2015), psychosocial circumstances (Howlin, 2000) and trying to ‘fit in’ (i.e. camouflaging; Hull et al., 2017). Ideally, many of these stressful situations should be reduced, for example, by improvements in diagnosis and health care, by improvements in education and employment opportunities, and inclusion and acceptance of autism in society. As stress is a part of life, making sure that at least individuals feel to be in control, this could have many beneficial effects and increasing this feeling is, therefore, worthwhile to pursuit.

## Supplemental Material

AUT872373_Lay_Abstract – Supplemental material for Autism and depression are connected: A report of two complimentary network studies Click here for additional data file. Supplemental material, AUT872373_Lay_Abstract for Autism and depression are connected: A report of two complimentary network studies by Barbara FC van Heijst, Marie K Deserno, Didi Rhebergen and Hilde M Geurts in Autism

AUT872373_Supplemental_material – Supplemental material for Autism and depression are connected: A report of two complimentary network studies Click here for additional data file. Supplemental material, AUT872373_Supplemental_material for Autism and depression are connected: A report of two complimentary network studies by Barbara FC van Heijst, Marie K Deserno, Didi Rhebergen and Hilde M Geurts in Autism

## Notes

1. We use ‘autism’ as the term for the full autism spectrum; classic autism, Asperger syndrome, PDD-NOS and autism spectrum disorder/condition.

2. One could argue that the similar reports on mastery and worries between the two clinical groups is due to the presence of autistic individuals who currently report to have a co-occurring depression in the autism group. Also in the depression group, we cannot exclude the possibility that some participants are meeting criteria for an autism diagnosis. While the potential impact of the latter cannot be tested within the current samples, we can exclude the autistic individuals with comorbid depression disorders from our combined group analyses (i.e. MANCOVA across Study 1 and Study 2). When doing so, we observed a similar pattern of findings as in the full group analyses. The only difference observed was that the impact of age on social worries was no longer significant (*p* = 0.123). Therefore, the lack of mastery and observed worries cannot be solely attributed to the inclusion of currently depressed autistic individuals.
