# Supplementary material for: Autism and depression are connected: A report of two complimentary network studies
Source: Autism. 2019 Nov 10;24(3):680–92. doi: 10.1177/1362361319872373 (PMC7168804; doi:10.1177/1362361319872373)
Supplement: AUT872373_Lay_Abstract – Supplemental material for Autism and depression are connected: A report of two complimentary network studies [file AUT872373_Lay_Abstract.pdf]

Lay Abstract

autism

DOI: 10.1177/1362361319872373

Autism and depression are connected: A report of two complimentary network studies

Barbara FC van Heijst, Marie K Deserno, Didi Rhebergen and Hilde M Geurts

Autistic people often experience depression. The goal of this paper was to test two specific hypothesis related to why autism and depression might co-occur: (1) some of the symptoms of autism and depression overlap; and (2) there is something that's not part of autism or depression itself, that connects the two conditions.

Autistic people and depressed people both experience lots of stress, which is why we looked at two important factors of coping with stress: (1) worrying; and (2) feeling like you are in control and can handle life's stressors (which is called perceived mastery). We looked at worry and perceived mastery as possible 'connectors' of autism and depression.

To study this, we looked at questionnaire responses from two different groups of people: Study 1 mainly included older adults with a diagnosis of depression; Study 2 mainly included middle aged and older adults with an autism diagnosis.

With a technique called network analysis we tested our two hypotheses.

The results showed that the co-occurrence of autism and depression could not be fully explained by overlapping symptoms.

We have to be careful with the interpretation of the results, but it seems like the second hypothesis might be right: perceived mastery, in particular, seems to be important in connecting autism and depression.

This is an important finding, as a low sense of mastery could signal vulnerability to developing mental health problems such as depression. Mastery is something that could be targeted in interventions designed to increase a person's wellbeing.
