# Supplementary material for: Autism and depression are connected: A report of two complimentary network studies
Source: Autism. 2019 Nov 10;24(3):680–92. doi: 10.1177/1362361319872373 (PMC7168804; doi:10.1177/1362361319872373)
Supplement: AUT872373_Supplemental_material – Supplemental material for Autism and depression are connected: A report of two complimentary network studies [file AUT872373_Supplemental_material.pdf]

Supplementary materials Autism and Depression Connected

Section 1:

Distribution of scores of the dependent measures for both groups separately (Figure A Depression, Figure B Autism).

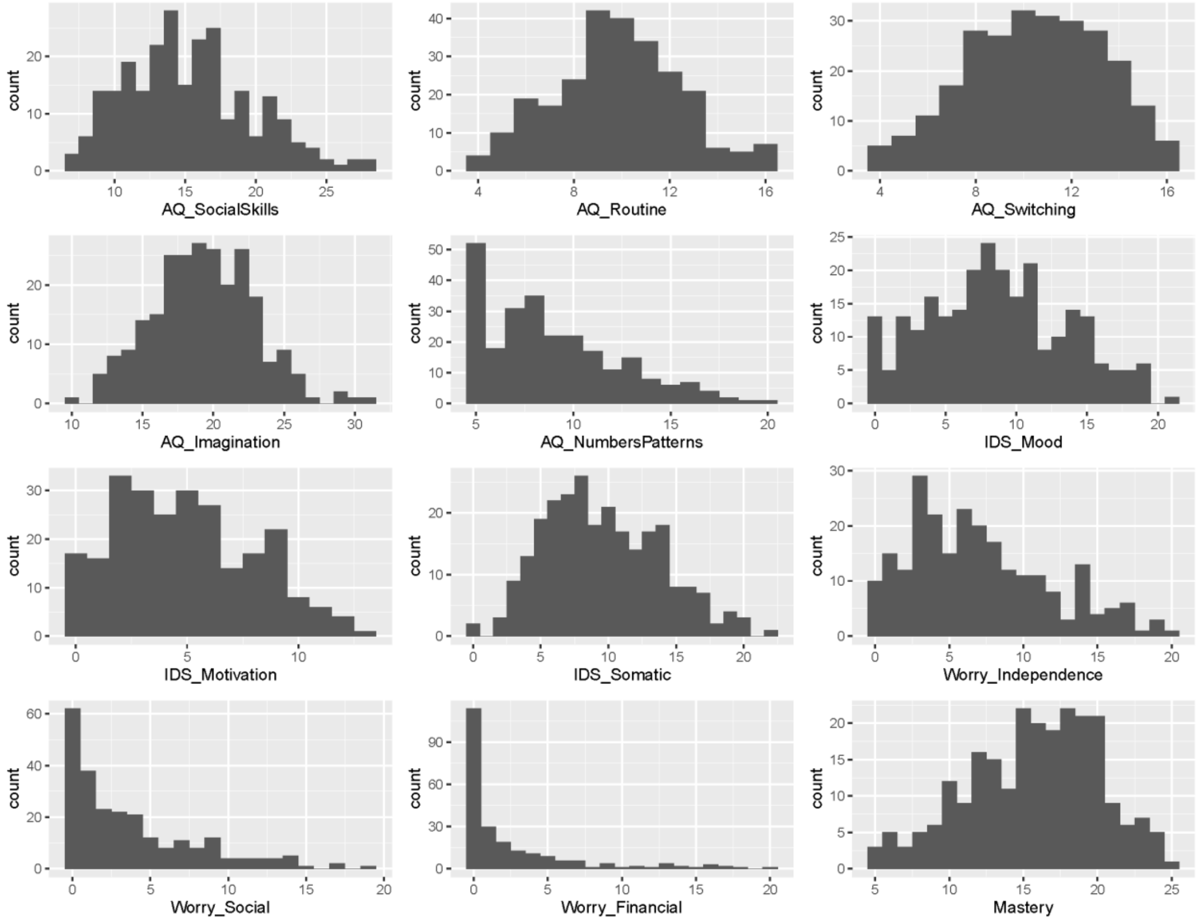

Figure A. Distribution of dependent measures in the depression cohort.

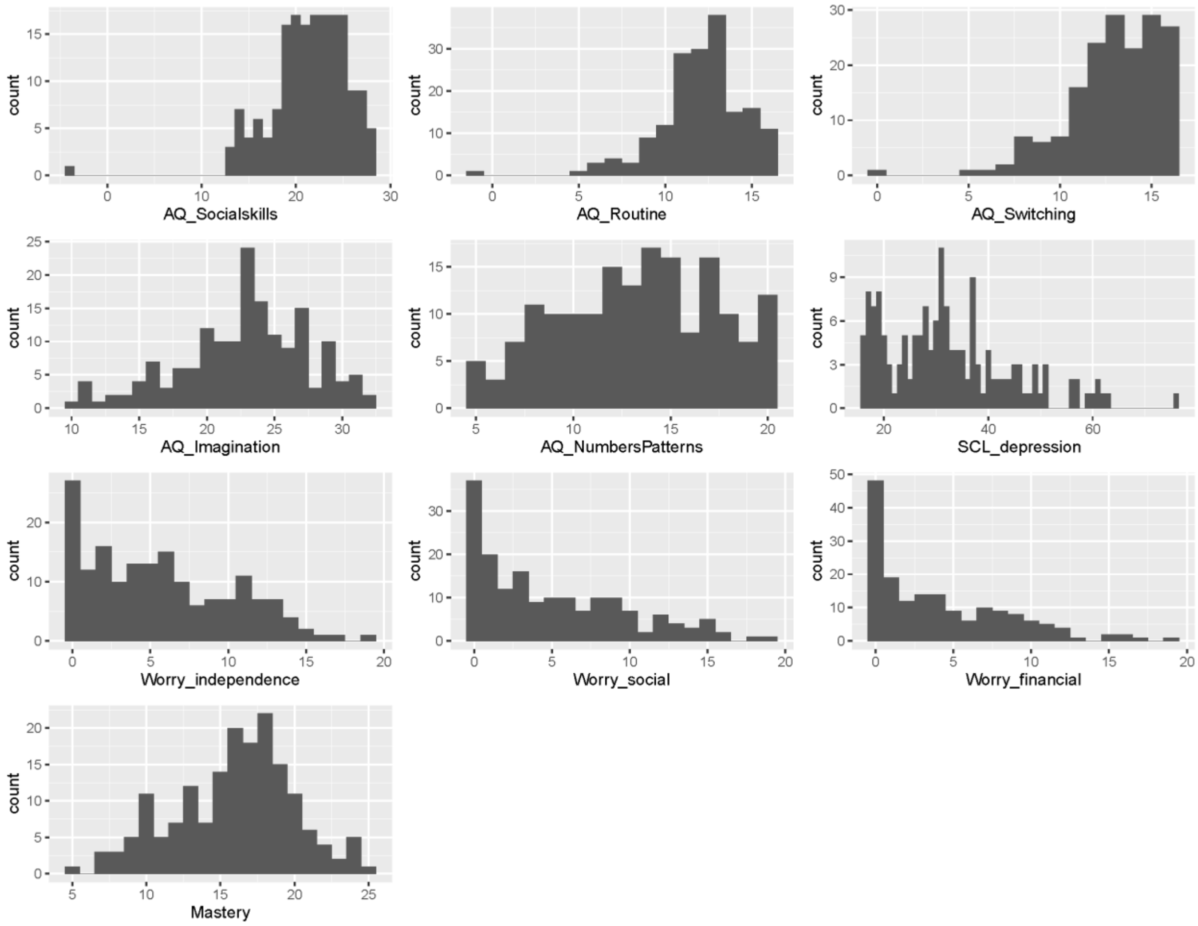

Figure B. Distribution of dependent measures in the autism cohort.

Section 2:

Results of the bootstrap of both the depression (Figure A) and autism cohort (Figure B). In the depression cohort, the stability centrality coefficients for betweenness is <.05 and for strength just above the .5 threshold, i.e. 0.52. For the autism cohort, stability centrality coefficients for both metrics are below the threshold (0.13 for betweenness and 0.44 for strength).

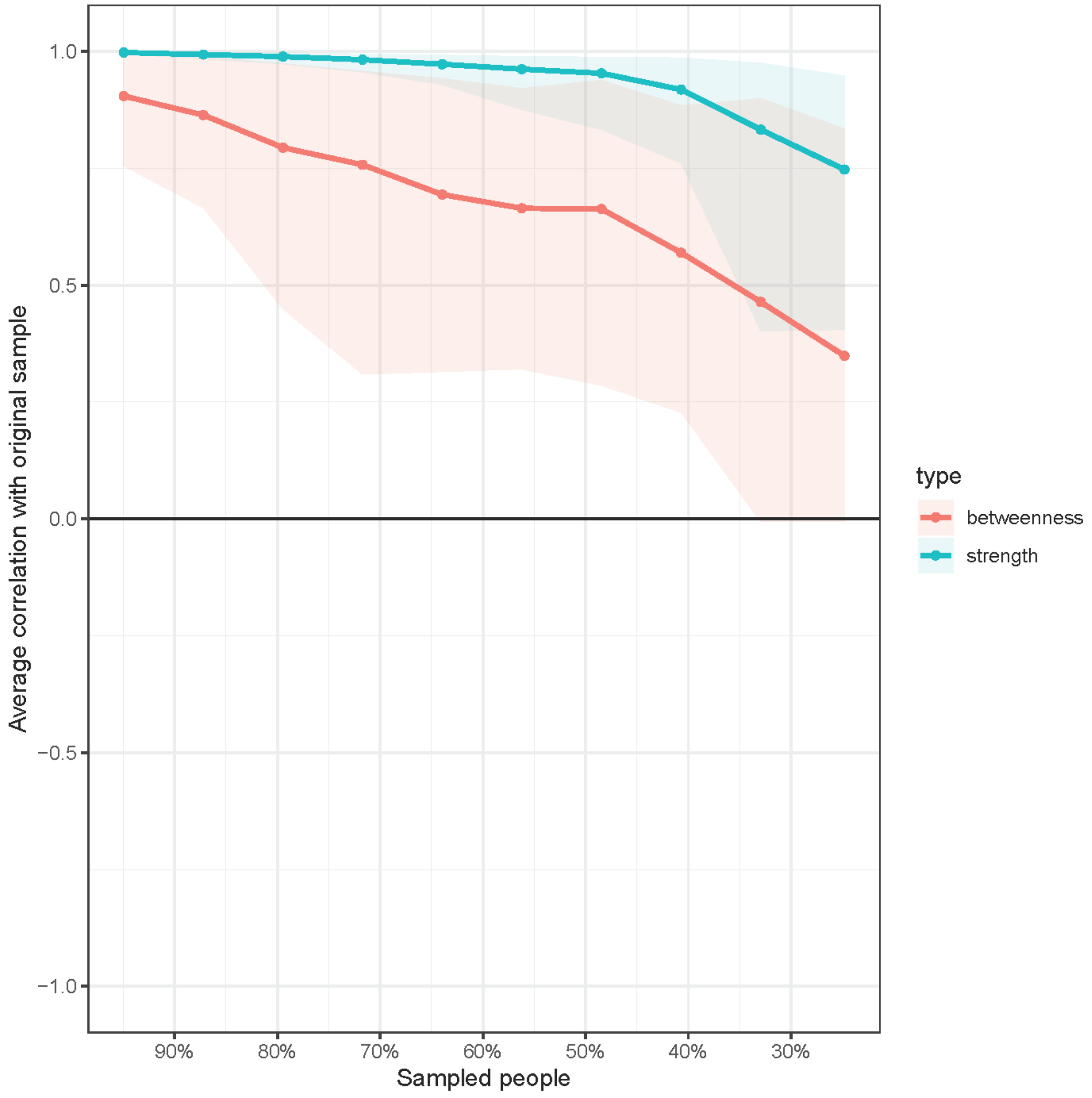

Figure A. Bootstrapping results of the stability of the metrics of the network analyses in the depression cohort.

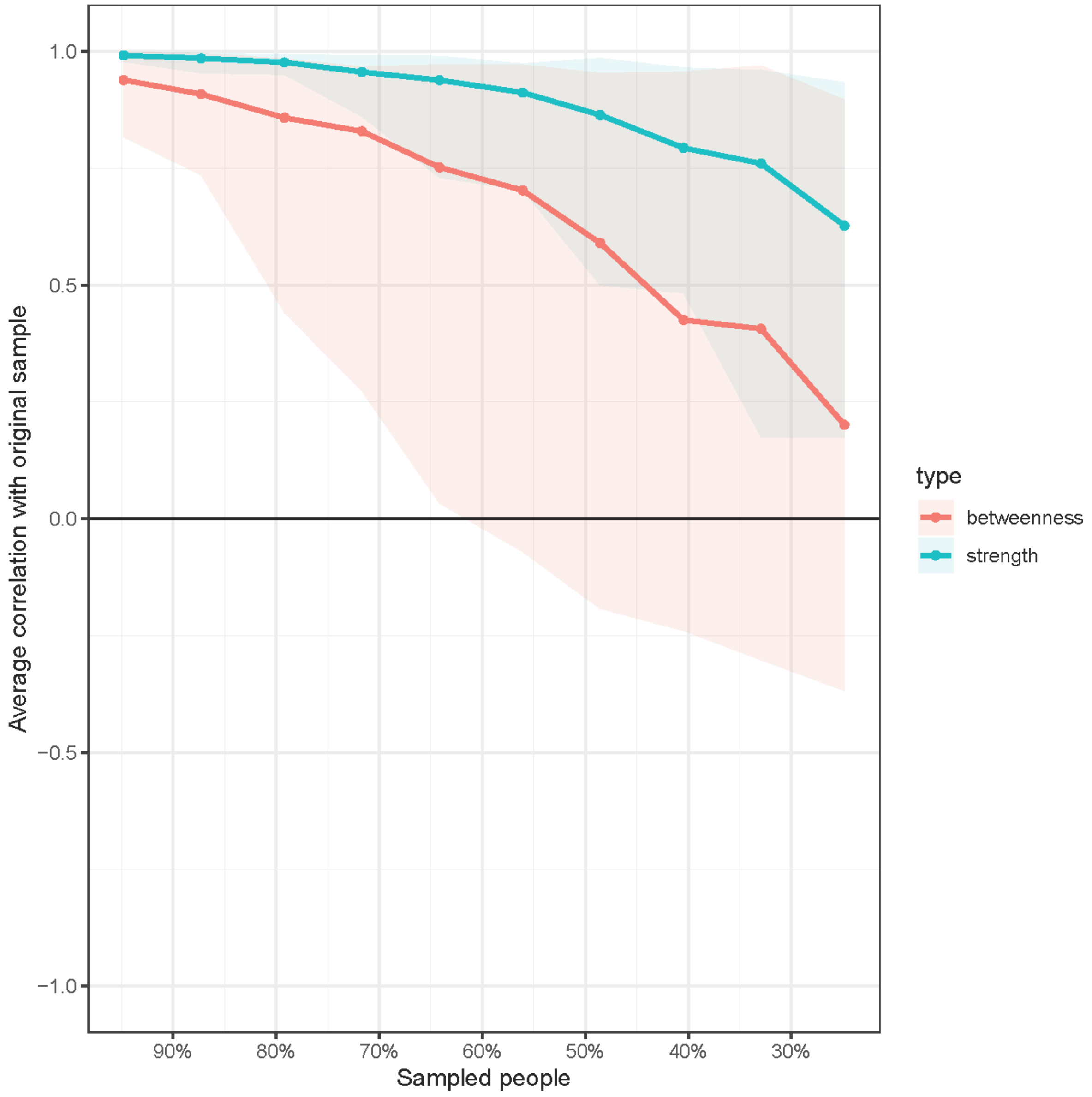

Figure B. Bootstrapping results of the stability of the metrics of the network analyses in the autism cohort.

Section 3:

Detailed results of the MANCOVA as described in results section of Study 2 in the manuscript

Table A. *Mean and standard deviation of the worry subscales and mastery total scale score for the two clinical groups and the combined comparison group.*

	Depression group (N=225)	Autism group (N=166)	Comparison group (N=176)
Indep	6.9 (4.8)	5.8 (4.5)	3.7 (3.7)
Social	3.7 (4.2)	4.9 (4.8)	0.8 (1.9)
Financial	2.5 (4.2)	4.1 (4. 4)	1.3 (2.7)
Mastery	15.6 (4.4)	15.9 (4.1)	9.0 (3.2)

Note. Worries measured by the Worry Scale-R: Indep = worries about loss of independence; Social = worries about social conditions; Financial = financial worries;. Mastery measured by the Pearlin Mastery Scale: Mastery.

Table B. *Pairwise comparisons of the worry subscales and mastery total scale score between the two clinical groups and the comparison group.*

Dependent Variable	(I) Groups	(J) Groups	Mean Difference (I-J)	Std. Error	Sig
Indep	Depression group	Autism group	1.25	.543	.063
	Comparison group	Depression group	-3.26	.455	.000
		Autism group	-2.00	.517	.000
Social	Depression group	Autism group	-0.67	.469	.395
	Comparison group	Depression group	-3.11	.393	.000
		Autism group	-3.78	.447	.000
Financial	Depression group	Autism group	-0.03	.463	1.00
	Comparison group	Depression group	-1.77	.387	.000
		Autism group	-1.80	.440	.000
Mastery	Depression group	Autism group	-0.10	.488	.996
	Comparison group	Depression group	-6.67	.408	.000
		Autism group	-6.77	.464	.000

Note. Worries measured by the Worry Scale-R: Indep = worries about loss of independence ,Social = worries about social conditions; Financial = financial worries. Mastery measured by the Pearlin Mastery Scale: Mastery.
